# m^6^A demethylase ALKBH5 suppression contributes to esophageal squamous cell carcinoma progression

**DOI:** 10.18632/aging.203490

**Published:** 2021-09-07

**Authors:** Dong Xiao, Ting-Xiao Fang, Ye Lei, Sheng-Jun Xiao, Jia-Wei Xia, Tao-Yan Lin, Yong-Long Li, Jian-Xue Zhai, Xiao-Yan Li, Shi-Hao Huang, Jun-Shuang Jia, Yu-Guang Tian, Xiao-Lin Lin, Kai-Can Cai, Yan Sun

**Affiliations:** 1 Laboratory Animal Center, Southern Medical University, Guangzhou 510515, China; 2 Guangzhou Southern Medical Laboratory Animal Sci. & Tech. Co., Ltd., Guangzhou 510515, China; 3 Guangzhou Key Laboratory of Tumor Immunology Research, Cancer Research Institute, School of Basic Medical Sciences, Southern Medical University, Guangzhou 510515, China; 4 Department of Thoracic Surgery, Nanfang Hospital, Southern Medical University, Guangzhou 510515, China; 5 National Demonstration Center for Experimental Education of Basic Medical Sciences, Southern Medical University, Guangzhou 510515, China; 6 Department of Pathology, The Second Affiliated Hospital, Guilin Medical University, Guilin 541199, China; 7 The Third People’s Hospital of Kunming, The Sixth Affiliated Hospital of Dali University, Kunming 650041, China; 8 Department of Pharmacy, Nanfang Hospital, Southern Medical University, Guangzhou 510515, China; 9 Zhongshan School of Medicine, Sun Yat-Sen University, Guangzhou 510080, China

**Keywords:** esophageal squamous cell carcinoma, m^6^A RNA modification, ALKBH5, cell proliferation, tumorigenicity

## Abstract

Esophageal squamous cell carcinoma (ESCC) is a highly malignant gastrointestinal cancer with a high recurrence rate and poor prognosis. Although N^6^-methyladenosine (m^6^A), the most abundant epitranscriptomic modification of mRNAs, has been implicated in several cancers, little is known about its participation in ESCC progression. We found reduced expression of ALKBH5, an m^6^A demethylase, in ESCC tissue specimens with a more pronounced effect in T3-T4, N1-N3, clinical stages III–IV, and histological grade III tumors, suggesting its involvement in advanced stages of ESCC. Exogenous expression of ALKBH5 inhibited the *in vitro* proliferation of ESCC cells, whereas depletion of endogenous ALKBH5 markedly enhanced ESCC cell proliferation *in vitro*. This suggests ALKBH5 exerts anti-proliferative effects on ESCC growth. Furthermore, ALKBH5 overexpression suppressed tumor growth of Eca-109 cells in nude mice; conversely, depletion of endogenous ALKBH5 accelerated tumor growth of TE-13 cells *in vivo*. The growth-inhibitory effects of ALKBH5 overexpression are partly attributed to a G1-phase arrest. In addition, ALKBH5 overexpression reduced the *in vitro* migration and invasion of ESCC cells. Altogether, our findings demonstrate that the loss of ALKBH5 expression contributes to ESCC malignancy.

## INTRODUCTION

Esophageal squamous cell carcinoma (ESCC) is a highly aggressive histological subtype of esophageal cancer reported in Asia, with China having one of the highest morbidity and mortality rates [1–3]. The mainstay of treatment includes surgical resection, radiotherapy, and chemotherapy [1–3]; however, these are associated with unsatisfactory clinical outcomes due to adverse effects and limited efficacy. A detailed understanding of ESCC immunobiology would be useful in developing efficient prognostic biomarkers and therapeutic targets that can detect the tumor at an early stage, consequently resulting in early diagnosis and treatment.

In mammals, m^6^A modification of mRNAs is catalyzed by a methyltransferase complex consisting of METTL3 and METTL14 (m^6^A writers) and removed by two independent demethylases, namely FTO and ALKBH5 (m^6^A erasers) [4–7]. The effect on target mRNAs depends on the activity of m^6^A-binding proteins (readers)—YTH domain-containing family proteins YTHDF1/2/3, YTHDC1, and YTHDC2; RNA-binding proteins—that have been implicated in several aspects of mRNA metabolism, including mRNA splicing, localization, translation, and decay [4–7]. The m^6^A modification on mRNAs regulates several physiological and pathological processes such as embryonic stem cell fate [8, 9], somatic cell reprogramming and pluripotency [10], hematopoietic stem/progenitor cell lineage specification and differentiation [11–13], axon regeneration [14], sex determination [15, 16], T cell homeostasis [17], innate immunity [18], DNA damage [19], and spermatogenesis [20].

Numerous studies have demonstrated the association of mRNA m^6^A modification with malignant progression of several tumors, including leukemia, lung cancer, breast cancer, colorectal cancer, hepatocellular carcinoma, glioma, prostate cancer, melanoma, endometrial cancer, ovarian cancer, and ESCC [4–7, 21–24]. For instance, upregulated expression of METTL3 in ESCC is associated with poor survival, making it an effective independent predictor of disease-free and overall survival of ESCC patients [24]. A recent study revealed that rs2416282, a single nucleotide polymorphism in the promoter of m^6^A reader YTHDC2, contributed to ESCC risk by regulating YTHDC2 expression [22]. Moreover, YTHDC2 knockdown substantially increased ESCC cell proliferation by affecting several cancer-related signaling pathways [22]. Similarly, upregulated expression of FTO in ESCC tissues is indicative of its oncogenic potential, as evident from increased proliferation and migration of ESCC cells [23].

ALKBH5 is implicated in various physiological and pathological processes including DNA damage [25], autophagy [26, 27], ferroptosis [28], innate immune response [29], brain development [30], cardiomyocyte proliferation and heart regeneration [31], ossification [32], osteogenic differentiation [33], systemic lupus erythematosus [34], diabetes [35], reproductive system diseases [36], rheumatoid arthritis [37], Hirschsprung's disease [38], recurrent miscarriage [39], and cancers [28, 40–49]. We investigated the effects of ALKBH5 on the proliferation, tumorigenicity, migration, and invasion of ESCC cells.

## RESULTS

### Reduced ALKBH5 expression is frequently detected in ESCC tissue

**Figure 1:**
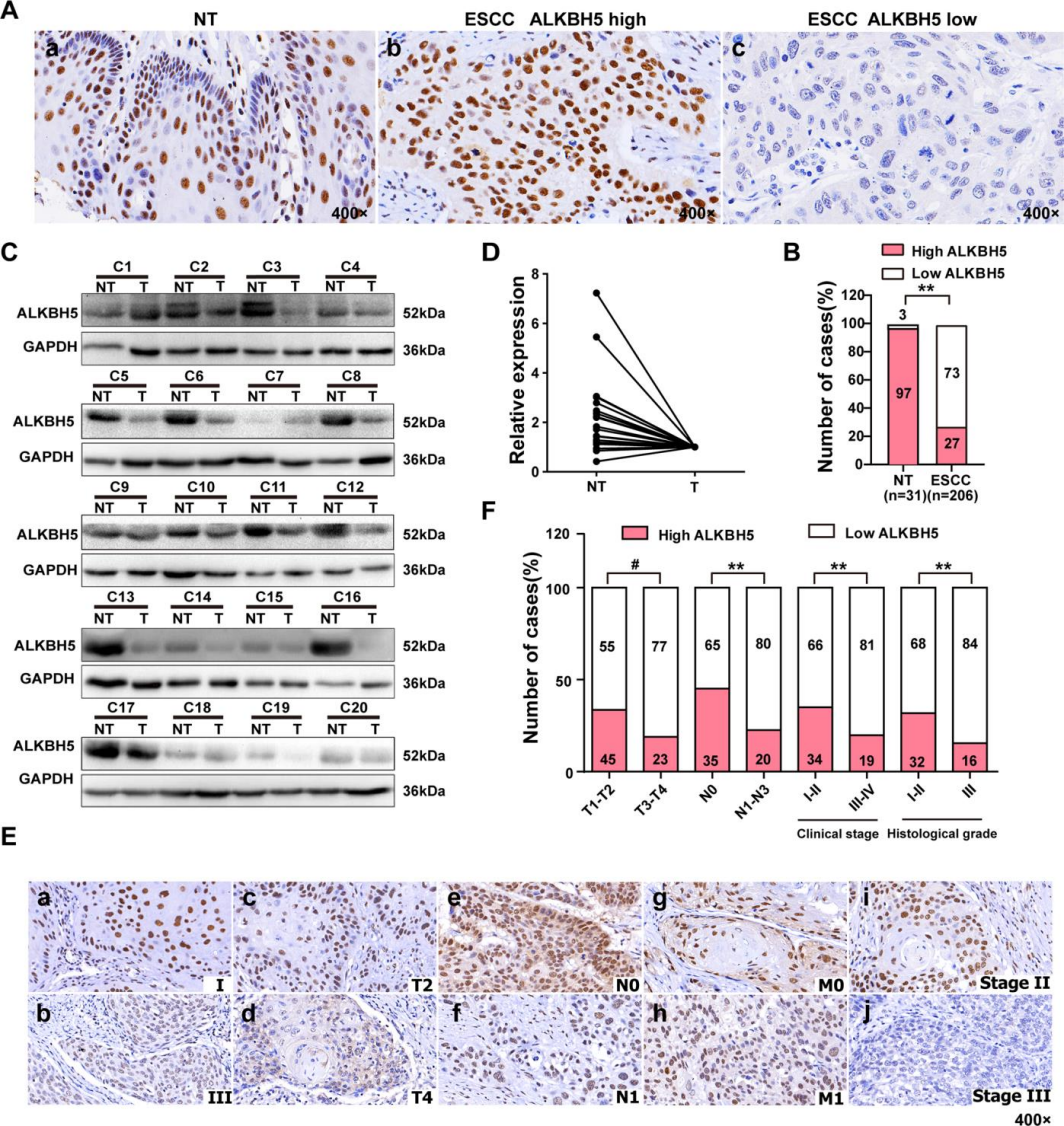
**ALKBH5 was downregulated in ESCC tissue specimens, and its downregulation was associated with advanced TNM and clinical stages of ESCC.** (**A**) Representative immunohistochemistry images showing ALKBH5 protein expression in adjacent non-tumor (NT) and ESCC tissue specimens. (**a**) High expression of ALKBH5 in adjacent non-tumor (NT) specimens. (**b**) High expression of ALKBH5 in ESCC specimens. (**c**) Low expression of ALKBH5 in ESCC specimens. The brown staining indicates ALKBH5 immunoreactivity. (**B**) IHC assay revealed lower ALKBH5 expression in ESCC tissue specimens than in the adjacent healthy tissues (*P* < 0.01, ᵡ^2^ test). (**C**) Western blots showing ALKBH5 protein expression in ESCC specimens and paired adjacent NT biopsies. (**D**) Western blotting revealed lower ALKBH5 expression in ESCC tissue specimens than in the adjacent healthy tissues. (**E**) Representative images of ALKBH5 expression in ESCC biopsies with different TNM and clinical stages. High expression of ALKBH5 was observed in clinical stages I (**a**), T2 (**c**), N0 (**e**), M0 (**g**) and histological grade II (**i**) of ESCC biopsies, whereas low expression of ALKBH5 was detected in clinical stages III (**b**), T4 (**d**), N1 (**f**), and M1 (**h**), and histological grade III (**j**). (**F**) Numbers and percentages of disease cases with high or low expression of ALKBH5 according to different clinicopathological features.

To study the involvement of ALKBH5 in ESCC progression, we first checked the expression of ALKBH5 protein in 206 paraffin-embedded, archived ESCC specimens and 31 adjacent non-cancerous tissues (NT) by immunohistochemistry (IHC). Low expression of ALKBH5 was detected in 1 NT sample (3%) (Figure 1A-a and Table 1) and 150 ESCC specimens (73%) (Figure 1A-b, c, 1B, and Table 1; *P* < 0.01). Western blotting revealed reduced ALKBH5 expression in 20 fresh ESCC specimens compared with adjacent non-cancerous tissues (Figure 1C, 1D). Furthermore, IHC revealed ALKBH5 protein to be primarily located in the nucleus of cancer cells (Figure 1A, 1E).

**Table 1:** Expression of ALKHB5 in 31 non-cancerous epithelial tissues and 206 ESC tissues.

**Variables**	***n***	**ALKBH5 expression**		**χ2**	***P***
		**Low (*n*, %)**	**High (*n*, %)**		
non-cancerous epithelial tissues	31	1(3)	30(97)	56.44	<0.001
ESCC	206	150(73)	56(27)		

### Reduced ALKBH5 expression is positively correlated with advanced ESCC

Table 2 shows the relationship between ALKBH5 expression and several clinicopathologic characteristics of ESCC patients. Although no association was identified between ALKBH5 expression and age (*P* = 0.524) and sex (*P* = 1.000) using 206 ESCC samples (Table 2), ALKBH5 expression was inversely correlated with tumor size (T classification; *P* = 0.006), lymph node invasion (N classification, *P* = 0.019), clinical stage (I–II versus III–IV, *P* = 0.027), and histological grade (*P* = 0.023) in these patients (Figure 1E, 1F and Table 2). Reduced expression of ALKBH5 was more frequently observed in T3–T4, N1–N3, clinical stage III–IV, and histological grade III tumors than in T1–T2, N0, clinical stage I–II, and histological grade I–II tumors (Figure 1E, 1F and Table 2), indicating ALKBH5 loss as a major molecular event in advanced cases of ESCC. Altogether, these results suggested the involvement of ALKBH5 in the progression of ESCC.

**Table 2:** Correlation between ALKHB5 expression and the clinicopathological features in 206 ESC patients.

**Characteristics**	**Case No.(n)**	**ALKBH5 expression**		***χ2***	**P**
		**High (n,%)**	**Low (n,%)**		
Sex					
Female	30	8(27)	22(73)	0.005	1.000
Male	176	48(27)	128(73)		
Age (years)					
<50	32	7(22)	25(78)	0.540	0.524
≥50	174	49(28)	125(72)		
Histological grade					
I + II	148	47(32)	101(68)	5.552	0.023
III	58	9(16)	49(84)		
T classification					
T1-T2	42	19(45)	23(55)	8.687	0.006
T3-T4	164	37(23)	127(77)		
N classification					
N0	100	35(35)	65(65)	5.997	0.019
N1-N3	106	21(20)	85(80)		
Clinical stage					
I - II	116	39(34)	77(66)	5.556	0.027
III-IV	90	17(19)	73(81)		

### ALKBH5 overexpression inhibits the proliferation of ESCC cells *in vitro*

**Figure 2:**
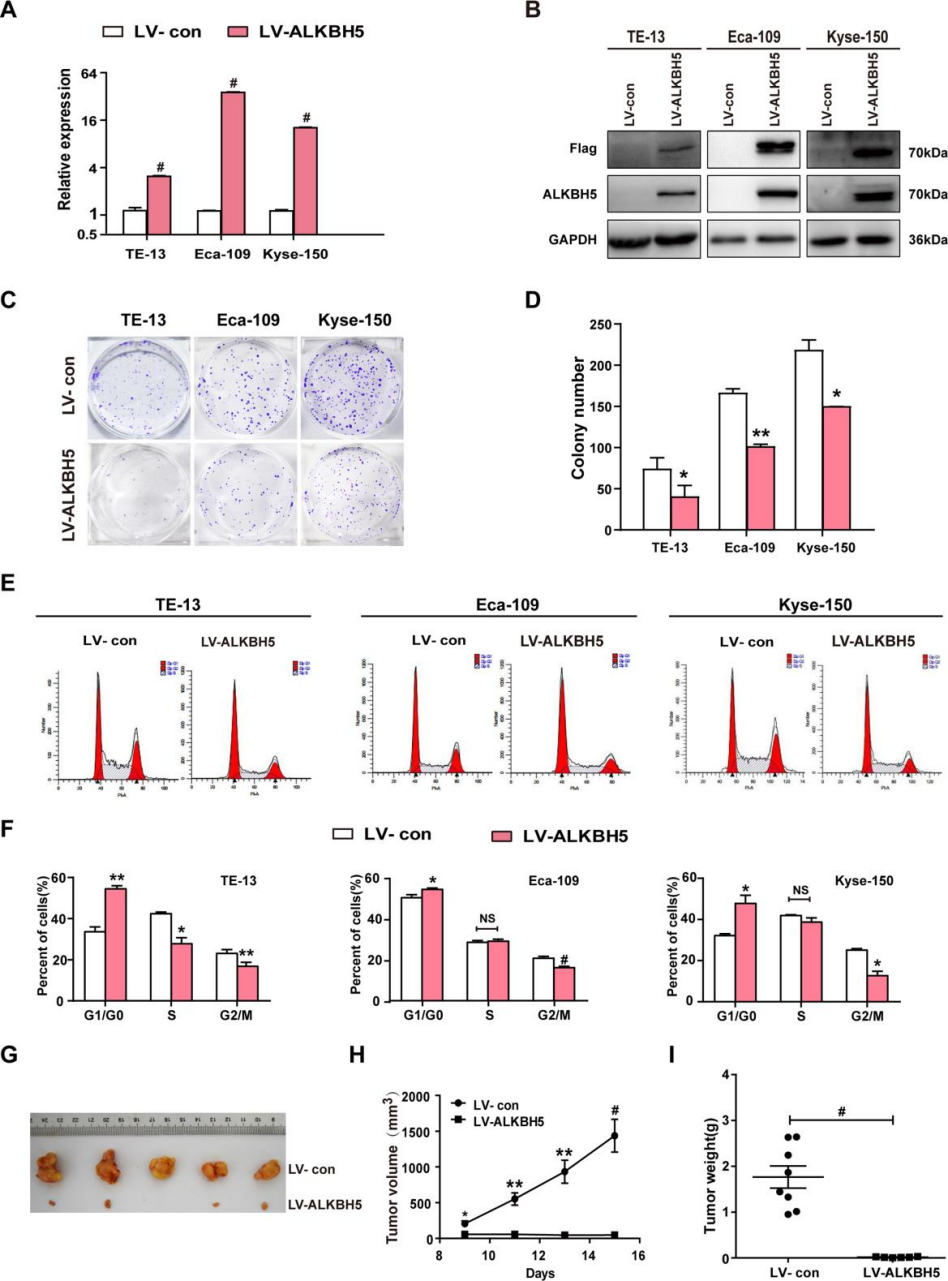
**ALKBH5 overexpression suppressed *in vitro* proliferation and *in vivo* tumorigenesis of ESCC cells.** (**A**, **B**) qRT-PCR (**A**) and western blot (**B**) analysis of ALKBH5 expression in vector-expressing (LV-con) and ALKBH5-expressing (LV-ALKBH5) ESCC cells (i.e., TE-13, Eca-109, and KYSE-150 cells). (**C**, **D**) A colony formation assay was performed to study the proliferation ability of vector- and ALKBH5-expressing ESCC cells. Left panels show representative images of colony formation assay (**C**) and right panels signify the total colony count (**D**). (**E**, **F**) Effects of ALKBH5 overexpression on cell cycle distribution in TE-13, Eca-109, and KYSE-150 cells. (**G**–**I**) ALKBH5 overexpression suppressed tumor growth of Eca-109 cells in nude mice. Vector- or ALKBH5-expressing Eca-109 cells were subcutaneously injected into the left and right dorsal thighs of mice, respectively. (**G**) Representative image of tumors formed. (**H**) Growth curve of tumor volumes. (**I**) Tumors were weighed.

The T classification data (Figure 1E, 1F and Table 2) revealed downregulated ALKBH5 in large-sized tumors, indicating an essential function of ALKBH5 in tumor growth. This observation prompted us to perform gain-of-function experiments to explore the effects of ALKBH5 on ESCC cell growth. Quantitative reverse transcriptase-polymerase chain reaction (qRT-PCR) and western blotting confirmed the successful overexpression of ALKBH5 transgene in TE-13, Eca-109, and KYSE-150 cells (Figure 2A, 2B). The colony formation assay showed that ALKBH5-expressing TE-13, Eca-109, and KYSE-150 cells displayed considerably fewer and smaller colonies compared with vector-expressing cells (Figure 2C, 2D; *P* < 0.01). These results suggested that re-expression of ALKBH5 markedly suppressed ESCC cell proliferation *in vitro*.

### RNAi-induced ALKBH5 silencing promotes ESCC cell proliferation *in vitro*

**Figure 3:**
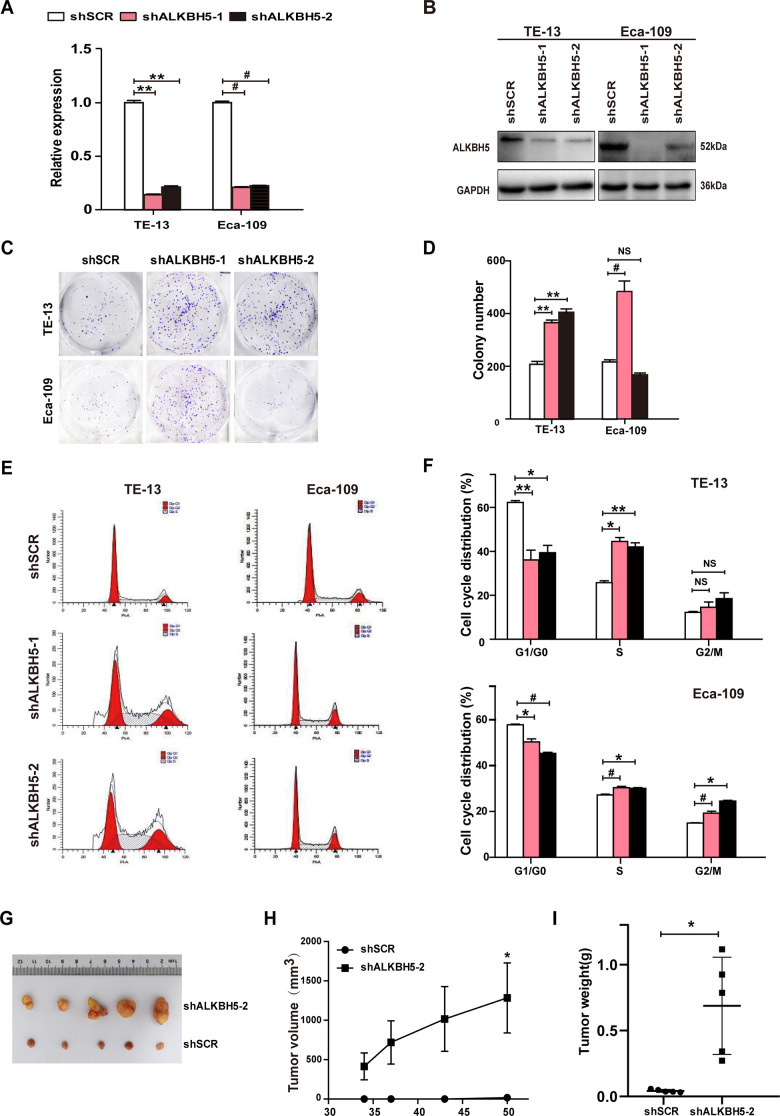
**RNAi-induced silencing of endogenous ALKBH5 promoted *in vitro* proliferation and *in vivo* tumorigenesis of ESCC cells.** (**A**, **B**) qRT-PCR (**A**) and western blot (**B**) analysis of ALKBH5 expression in shSCR-expressing (LV-shSCR) and shALKBH5-expressing (LV-shALKBH5-1 and LV-shALKBH5-2) ESCC cells (TE-13 and Eca-109 cells). (**C**, **D**) A colony formation assay was performed to study the proliferation ability of shSCR- and shALKBH5-expressing ESCC cells. Left panels show representative images of colony formation assay (**C**) and right panels signify the total colony count (**D**). (**E**, **F**) Effects of RNAi-induced ALKBH5 silencing on cell cycle distribution in TE-13 and Eca-109 cells. (**G**–**I**) ALKBH5 knockdown enhanced tumor growth of TE-13 cells in nude mice. shSCR- or shALKBH5-expressing TE-13 cells were subcutaneously injected into the left and right dorsal thighs of mice, respectively. (**G**) Representative image of tumors formed. (**H**) Growth curve of tumor volumes. (**I**) Tumors were weighed.

Because ALKBH5 was downregulated in ESCC tissue specimens (Figure 1A–1D and Table 1), we proposed that the loss of ALKBH5 expression was associated with ESCC progression. Thus, we next performed loss-of-function experiments to further examine the effects of loss of ALKBH5 function on ESCC cell growth. Both shRNA-ALKBH5-1 and shRNA-ALKBH5-2 specifically knocked down the expression of endogenous ALKBH5 mRNA (Figure 3A) and protein (Figure 3B) in both TE-13 and Eca-109 cells. The clonogenic assay revealed that shALKBH5-expressing TE-13 and Eca-109 cells formed larger colonies compared with shSCR-expressing cells (Figure 3C, 3D; *P* < 0.01 or 0.001). In summary, these findings showed that the loss of ALKBH5 expression promoted the growth of ESCC cells *in vitro*.

### Ectopic expression of ALKBH5 suppresses *in vivo* tumorigenicity of ESCC cells

To further examine the growth-inhibiting effects of ALKBH5 on ESCC cells *in vivo*, we performed subcutaneous tumor xenograft experiments in nude mice. Vector- and ALKBH5-expressing Eca-109 cells or shSCR- and shALKBH5-expressing TE-13 cells were subcutaneously injected into the dorsal flank of nude mice. The tumor size (Figure 2G), tumor volume (Figure 2H), and tumor weight (Figure 2I) were noticeably larger in tumors induced by vector-expressing cells compared with those induced by ALKBH5-expressing cells. On the contrary, the depletion of endogenous ALKBH5 remarkably accelerated tumor growth *in vivo* (Figure 3G–3I). Altogether, these results demonstrated that ALKBH5 negatively regulated the *in vivo* tumorigenicity of ESCC cells.

### ALKBH5 inhibits the G1-S phase transition of ESCC cells

Based on the growth inhibitory properties of ALKBH5, we next examined the cell-cycle distribution of vector- and ALKBH5-expressing ESCC cells. Compared with vector control, TE-13, Eca-109, and KYSE-150 cells overexpressing ALKBH5 displayed an increased percentage of cells in the G1/G0 phase and fewer cells in S and G2/M phases (Figure 2E, 2F; *P* < 0.05 or 0.01). Our results suggested that growth-suppressive effects of ALKBH5 overexpression were partly ascribed to a G1-phase arrest.

To further study the mechanism of ALKBH5 silencing in promoting ESCC cell growth, we analyzed the cell-cycle distribution using propidium iodide (PI) staining. As shown in Figure 3E, 3F, ALKBH5 depletion in TE-13 and Eca-109 cells markedly decreased the proportion of G1/G0 phase cells, and simultaneously increased the proportion of S phase cells. Thus, ALKBH5 silencing promoted the G1-S phase transition of ESCC cells. Altogether, these findings showed that ALKBH5 altered the cell cycle distribution of ESCC cells.

### ALKBH5 overexpression dramatically reduces the migration and invasion ability of ESCC cells

**Figure 4:**
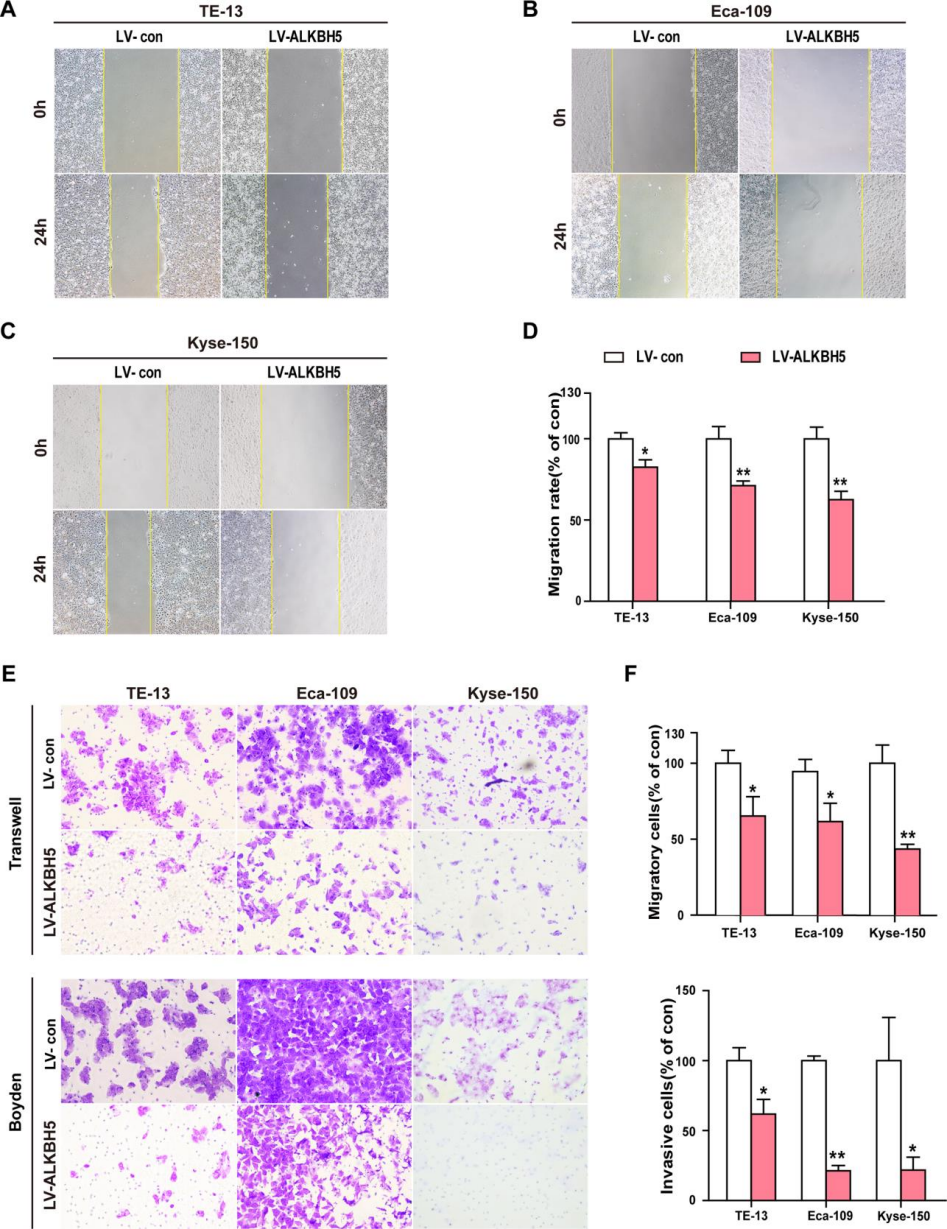
**Ectopic expression of ALKBH5 inhibited the migration and invasion of ESCC cells *in vitro*.** (**A**–**D**) Wound healing assays were performed in ALKBH5-expressing ESCC cells. The migration ability was determined by measuring the distance from the boundary of the scratch created to the cell-free space after 24 h. (**E**, **F**) The migratory and invasive activities of ALKBH5-expressing ESCC cells based on transwell migration and Boyden invasion assays, respectively. The average number of cells per field was calculated from three independent experiments (original magnification: ×200).

To study whether ALKBH5 overexpression directly suppressed the invasion and migration ability of ESCC cells, we examined the phenotypic changes in ESCC cells following ectopic expression of ALKBH5. Wound-healing assays showed that exogenous expression of ALKBH5 reduced the migration of TE-13, Eca-109, and KYSE-150 cells (Figure 4A–4D). As indicated in Figure 4E, 4F, Transwell and Boyden assays showed increased migration and invasion abilities of ALKBH5-expressing TE-13, Eca-109, and KYSE-150 cells as compared with vector-expressing cells. Altogether, these findings suggested that ALKBH5 overexpression inhibited the migration and invasion of ESCC cells *in vitro*.

### ALKBH5 silencing enhances ESCC cell migration and invasion *in vitro*

**Figure 5:**
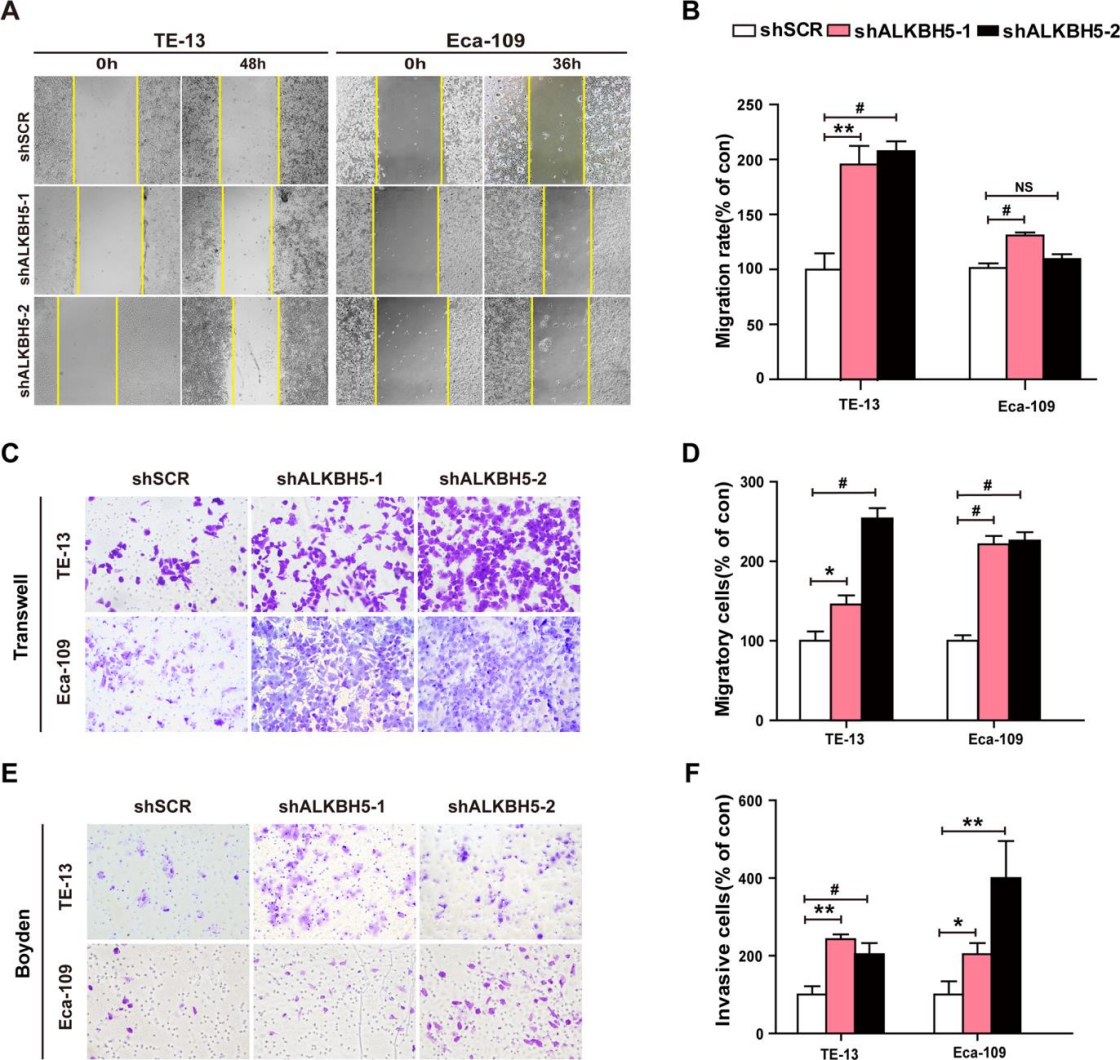
**RNAi-induced ALKBH5 silencing enhanced the migration and invasion of ESCC cells *in vitro*.** (**A**, **B**) Wound healing assays were performed in shALKBH5-expressing ESCC cells. The migration ability was determined by measuring the distance from the boundary of the scratch to the cell-free space after 36 h and 48 h. (**C**, **D**) The migration ability of shALKBH5-expressing ESCC cells using the transwell migration assay. The average number of cells per field was calculated from three independent experiments (original magnification: ×200). (**E**, **F**) The invasive ability of shALKBH5-expressing ESCC cells using the Boyden invasion assay. The average number of cells per field was calculated from three independent experiments (original magnification: ×200).

Because ALKBH5 was frequently under-expressed in N1-N3 tumors than in N0 tumors (Figure 1E, 1F and Table 2), we next explored the effects of ALKBH5 knockdown on cell migration and invasion of ESCC cells. Wound-healing assays demonstrated that ALKBH5 silencing promoted the migration of both TE-13 and Eca-109 cells (Figure 5A, 5B). Transwell migration and Boyden invasion assays showed that the shRNA-induced knockdown of endogenous ALKBH5 dramatically promoted the migration (Figure 5C, 5D) and invasion (Figure 5E, 5F) of TE-13 and Eca-109 cells. Altogether, the suppression of endogenous ALKBH5 expression enhanced the migration and invasion of ESCC cells.

## DISCUSSION

In mammalian cells, m^6^A writers (METTL3, and METTL14) and m^6^A erasers (FTO and ALKBH5) catalyze the methylation and demethylation of m^6^A modification of mRNAs, respectively [4–7, 21]. In addition, m^6^A reader proteins (YTHDF1/2/3, YTHDC1, and YTHDC2; RNA-binding proteins) selectively recognize and bind to m^6^A-methylated mRNAs at m^6^A sites and decide the fate of modified target mRNAs [4–7, 21]. These three categories of m^6^A regulatory proteins have been associated with malignant progression of various cancers, including leukemia, lung cancer [4–7, 21], breast cancer, colorectal cancer, hepatocellular carcinoma, glioma, prostate cancer, melanoma, endometrial cancer, and ovarian cancer. However, their functions in ESCC have been relatively less investigated [22–24]. We demonstrated the downregulated expression of ALKBH5 in human ESCC tissue specimens using IHC and western blotting. Furthermore, ALKBH5 expression inversely correlated with the tumor size, lymph node invasion, clinical stage, and histological grade in ESCC patients. ALKBH5 negatively regulated the proliferation, tumorigenicity, migration, and invasion of ESCC cells. These findings implied that ALKBH5 functions as a tumor suppressor in ESCC progression.

Depending on the cell type, ALKBH5 exhibits divergent functions in cancers. For example, ALKBH5 functions as a tumor suppressor in ESCC (this study), hepatocellular carcinoma [40], pancreatic cancer [43, 46], and lung cancer [44], whereas it exerts pro-tumorigenic effects on leukemia [45, 50], glioblastoma [51], and breast cancer [52]. ALKBH5 is known to suppress malignant hepatocellular carcinoma cells by epigenetically inhibiting LYPD1 via m^6^A modification [40]. ALKBH5 overexpression repressed pancreatic cancer tumorigenesis by reducing WIF-1 RNA methylation and inactivating Wnt signaling [46]. Furthermore, its overexpression abolished m^6^A-YTHDF2 modification and post-transcriptionally activated PER1 to hinder the progression of pancreatic cancer [43]. Its antiproliferative and anti-metastatic functions in lung cancer are attributed to YTHDF-dependent reduced YAP expression [44]. In contrast, hypoxia induces m^6^A-demethylation of NANOG mRNA in a HIF- and ALKBH5-dependent manner to cause breast cancer stem cell phenotype [52]. Similarly, ALKBH5 contributes to lung cancer cell proliferation and metastasis following intermittent hypoxia by inducing the expression of FOXM1 protein in an m^6^A-dependent manner [53]. ALKBH5-induced overexpression of FOXM1 contributes to the tumorigenicity of glioblastoma stem-like cells [51]. In addition, ALKBH5 is known to post-transcriptionally regulate TACC3 to promote tumorigenesis and cancer stem cell self-renewal in acute myeloid leukemia (AML) [45], whereas the KDM4C-ALKBH5-AXL signaling axis participates in chromatin alterations in AML leukemia stem cells [50]. Moreover, the ectopic expression of ALKBH5 activates the EGFR-PIK3CA-AKT-mTOR signaling pathway and stabilizes BCL 2 mRNA to prevent autophagy of epithelial ovarian cancer cells [26].

Promoter hypermethylation of tumor suppressor genes is frequently reported in cancer cells [54, 55]. We identified ALKBH5 as a tumor suppressor, whose expression was dramatically diminished in human ESCC clinical specimens. Further, its ability to prevent proliferation, tumorigenicity, migration, and invasion of ESCC cells confirmed its tumor-suppressive potential in ESCC cells, executed via epigenetic silencing of *ALKBH5* promoter.

In conclusion, ALKBH5 functions as a tumor suppressor in the pathogenesis of ESCC. We believe ALKBH5 can be a promising therapeutic target for advanced ESCC. Further functional studies, using a combination of MeRIP-seq or miCLIP-seq and RNA-seq assays, to demonstrate the involvement of m^6^A-modified mRNAs in ESCC are warranted to validate its therapeutic potential.

## MATERIALS AND METHODS

### Clinical specimens

This study was performed using paired ESCC and adjacent non-cancerous tissue samples (*n* = 20) obtained from the Department of Thoracic Surgery, Nanfang Hospital, Southern Medical University, Guangzhou, China. The study was conducted as per the protocols approved by the institutional review board of the Second Affiliated Hospital of Guilin Medical University and Southern Medical University and complied with patient data safety guidelines. Informed consent was obtained from the patients. The inclusion criteria for the study were: (1) pathological diagnosis of ESCC without metastasis to distant organs; (2) no anticancer therapy before surgery; (3) matched healthy tissue samples (obtained from an area more than 5 cm from the tumor lesion margin) and absence of tumor cells in healthy tissues as confirmed by histopathological examination. The Edmondson–Steiner (E–S) grading system was used for the histological grading of tumors.

A tissue chip consisting of formalin-fixed, paraffin-embedded 206 ESCC tissues and corresponding adjacent tissue punches was provided by Guilin Fanpu Biotechnology Co., Ltd. These samples were obtained between 2006 and 2010 from patients with primary ESCC and age ranging from 28 to 78 years (mean = 58.5 years) at diagnosis. Other information collected included patients’ histopathologic and raw survival data.

### Histological analysis and immunohistochemistry

Samples (human tumor xenografts established in nude mice, human ESCC clinical specimens, and adjacent healthy tissues) were fixed in 4% paraformaldehyde (PFA), prepared in a phosphate buffer, overnight at 4° C. The samples were embedded in paraffin, sectioned into 5 mm thick pieces, mounted on slides, dewaxed, and deparaffinized. Hematoxylin and eosin staining (H&E staining) was performed as per the standard protocols.

For immunohistochemical staining, samples were deparaffinized and rehydrated. Afterward, sections were treated under high pressure in a citrate buffer (pH 6.0) for 2 min for antigen retrieval. Bovine serum albumin (1%) and H_2_O_2_ (15 min at room temperature) were used to inhibit non-specific staining and quench endogenous peroxidase activity, respectively. Next, the sections were incubated with anti-ALKBH5 antibody (Sigma, HPA007196) overnight at 4° C. PBS served as the negative control. Subsequently, the sections were incubated with secondary antibody conjugated to HRP. The complex was visualized with DAB and counterstained with hematoxylin.

We referred to published standards for defining the low and high expression of ALKBH5 [56–58]. Tumor cells were graded depending on the staining intensity: 0 = no staining; 1= poor staining; 2 = moderate staining; and 3 = strong staining. Further, positive staining ratio, defined as ALKBH5-positive tumor cells/total number of tumor cells × 100%, was graded as 0 = negative tumor cells; 1 = less than 10% positive; 2 = 10–50% positive; and 3 = more than 50% positive. The positive staining grade was multiplied by intensity grade (0, 1, 2, 3, 4, 6, and 9) to obtain the staining results. Grades of ≦4 and ≧6 were regarded as low and high expression, respectively. All grading was performed blindly by two pathologists.

### Cell lines and cell culture

Human ESCC cell lines, namely TE-13, KYSE-150, and Eca-109, were a kind gift from Prof. Jun Li (Zhongshan School of Medicine, Sun Yat-Sen University). HEK293T cell line was procured from the American Type Culture Collection (ATCC). All cell lines were maintained in Dulbecco’s modified Eagle’s medium (DMEM)(Corning) supplemented with 10% fetal bovine serum (FBS) (Biological Industries) at 37° C in a humidified incubator containing 5% CO_2_.

### Plasmids, lentivirus production, and lentiviral transduction for generating stable cell lines

Lentiviral short-hairpin RNA (shRNA) constructs for human ALKBH5 were generated using the pLKO.1-puro cloning vector (Addgene, Cambridge, MA, USA) following manufacturer’s instructions. Oligonucleotides used for human ALKBH5 were shALKBH5-1, GAAAGGCTGTTGGCATCAATA and shALKBH5-2, CCTCAGGAAGACAAGATTAGA. ALKBH5 lentiviral expression plasmids (referred to as pLV-ALKBH5) were constructed by cloning the full-length open reading frames (ORFs) of human *ALKBH5* gene (NM_017758.3) into the lentiviral expression plasmid pEX-NEG-Lv183 (GeneCopoeia, Guangzhou, China). To produce lentiviruses, 293T cells were co-transfected with lentiviral vectors and lentiviral packaging plasmids psPAX2 and pMD2G (Addgene) as previously described [53]. Finally, the ESCC cells were infected with these lentivirus particles.

### RNA isolation and quantitative real-time PCR

Total RNA was isolated and reverse transcribed, following which qRT-PCR was performed as previously described [59]. The following primers were used: ALKBH5 forward primer: 5’-CGGCGAAGGCTACACTTACG-3’; ALKBH5 reverse primer: 5’-CCACCAGCTTTTGGATCACCA-3’; GAPDH forward primer: 5’- ACCCAGAAGACTGTGGATGG-3’; and GAPDH reverse primer: 5’-TCTAGACGGCAGGTCAGGTC-3’. GAPDH was used as the reference gene against which all samples were normalized. Relative fold change was calculated using the 2^- ΔΔCt^ method.

### Western blotting

Total protein was isolated and the lysate was prepared. The proteins were separated using sodium dodecyl sulfate-polyacrylamide gel electrophoresis (SDS-PAGE). The separated proteins were transferred to a polyvinylidene difluoride (PVDF) membrane. The blots were incubated with anti-GAPDH (Proteintech, 1:1000) or anti-ALKBH5 (Sigma, HPA007196, 1:1000) antibody, followed by incubation with HRP-labeled secondary antibodies. Enhanced chemiluminescence (ECL) (Cat. No: KGP1122, KeyGen Biotech) was used to visualize the protein bands. β-actin was used as a loading control.

### Colony formation assay

For the clonogenic assay, 500 cells/well were plated in 6-well plates and grown for 10 to 12 days. Afterward, colony formation assay was performed using a previously described method [59].

### Cell cycle analysis

For cell cycle analysis, 2 × 10^5^ cells per well were plated in 6-well plates. The cells were stained with PI and cell cycle distribution was studied using flow cytometry [59, 60].

### Tumor xenografts in animals

Male BALB/c nude mice (3–4 weeks old) were purchased from the Medical Laboratory Animal Center of Guangdong Province and were fed autoclaved water and laboratory rodent chow. Vector- or ALKBH5-expressing Eca-109 cells (1.5 × 10^7^ cells) or shSCR- or shALKBH5-expressing TE-13 cells (1.5 × 10^7^ cells) were subcutaneously injected into the left or right dorsal thigh of the mice, respectively. The animals were monitored daily, and tumor volumes were measured using a caliper. Tumor volume was calculated using the following formula: volume = 1/2 (width^2^ × length) 0.5 × width^2^ × length. All animals were sacrificed on the 14th day after transplantation. All animal experiments were performed in strict accordance with the recommendations in the Guide for the Care and Use of Laboratory Animals and were approved by the Ethics Committee of the Southern Medical University.

### Wound healing assay

The cells were grown to near confluence in 6-well plates, followed by starvation in a serum-free medium for 24 h. A wound was created on the cell monolayer using a sterile 200 μL microtip. The cells were again starved for 48 h. The cells were observed under an inverted microscope (Nikon, Japan), and images of scratch areas were captured at 0, 24, 36, and 48 h to study the migration of cells to the wound area.

### Transwell migration and Boyden invasion assays

Transwell migration and Boyden invasion assays were performed using the methods described previously. For the transwell migration assay, vector- or ALKBH5-expressing ESCC cells (2 × 10^5^) or shSCR- or shALKBH5-expressing ESCC cells (2 × 10^5^) were seeded into the upper chamber of the transwell insert (Corning) with serum-free DMEM. For the Boyden invasion assay, the upper chamber was coated with Matrigel (BD Biosciences). DMEM with 10% FBS was added to the lower compartment as a chemoattractant. Cells were allowed to migrate for 17 h and 20 h in the transwell migration and Boyden invasion assays, respectively.

### Statistical analysis

Data from three independent experiments are presented as mean ± standard deviation (SD). Statistical analysis was performed using SPSS 16.0. Statistical significance was assessed by Student’s *t*-test (*P < 0.05, **P < 0.01, and ^#^*P* < 0.001; NS: not significant).
